# 4,4′-Diethyl-2,2′-[(*N*-cyclo­hexyl­imino)­bis­(methyl­ene)]diphenol

**DOI:** 10.1107/S1600536812040342

**Published:** 2012-09-29

**Authors:** Worawat Wattanathana, Chatchai Veranitisagul, Attaphon Kaewvilai, Apirat Laobuthee, Nattamon Koonsaeng

**Affiliations:** a Department of Chemistry, Faculty of Science, Kasetsart University, Bangkok 10900, Thailand; b Department of Materials and Metallurgical Engineering, Faculty of Engineering, Rajamangala University of Technology Thanyaburi, Pathumthani 12110, Thailand; c Department of Materials Engineering, Faculty of Engineering and Center of Advanced Studies in Industrial Technology, Kasetsart University, Bangkok 10900, Thailand

## Abstract

The title compound, C_24_H_33_NO_2_, exhibits an intra­molecular hydrogen bond between a phenol –OH group and the N atom. In the crystal, mol­ecules are connected by pairs of O—H⋯O hydrogen bonds.

## Related literature

For details of the synthesis of *N*,*N*-bis­(2-hy­droxy­benz­yl)alkyl­amines, see: Laobuthee *et al.* (2003 ▶). For their metal-responsive properties, see: Veranitisagul *et al.* (2011 ▶). For their use in the synthesis of macrocyclic mol­ecules, see: Rungsimanon *et al.* (2008 ▶).
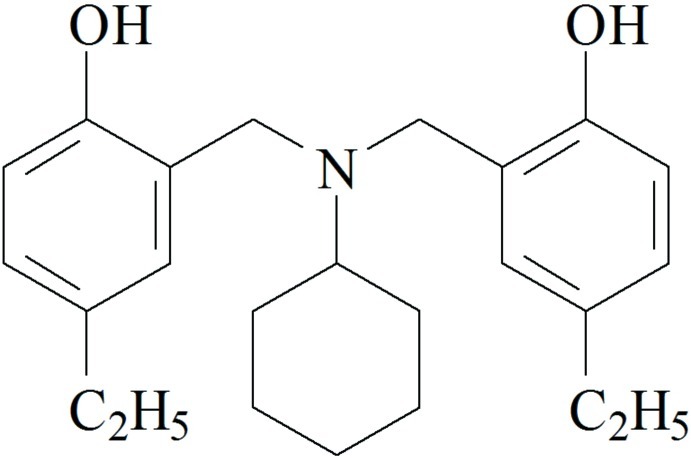


## Experimental

### 

#### Crystal data

C_24_H_33_NO_2_
*M* *_r_* = 367.51
Triclinic, 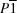

*a* = 9.4224 (12) Å
*b* = 10.3799 (16) Å
*c* = 11.8143 (18) Å
α = 82.140 (4)°
β = 73.960 (4)°
γ = 81.676 (4)°
*V* = 1093.1 (3) Å^3^
*Z* = 2
Mo *K*α radiation
μ = 0.07 mm^−1^
*T* = 296 K
0.90 × 0.44 × 0.24 mm

#### Data collection

Bruker APEXII CCD diffractometer
8721 measured reflections
5350 independent reflections
3648 reflections with *I* > 2σ(*I*)
*R*_int_ = 0.022

#### Refinement

*R*[*F* ^2^ > 2σ(*F* ^2^)] = 0.060
*wR*(*F* ^2^) = 0.185
*S* = 1.12
5350 reflections
252 parameters
H atoms treated by a mixture of independent and constrained refinement
Δρ_max_ = 0.27 e Å^−3^
Δρ_min_ = −0.23 e Å^−3^

### 

Data collection: *APEX2* (Bruker, 2007 ▶); cell refinement: *SAINT* (Bruker, 2007 ▶); data reduction: *SAINT*; program(s) used to solve structure: *SHELXS97* (Sheldrick, 2008 ▶); program(s) used to refine structure: *SHELXL97* (Sheldrick, 2008 ▶); molecular graphics: *SHELXTL* (Sheldrick, 2008 ▶); software used to prepare material for publication: *SHELXL97* and *publCIF* (Westrip, 2010 ▶).

## Supplementary Material

Crystal structure: contains datablock(s) I, global. DOI: 10.1107/S1600536812040342/gg2100sup1.cif

Structure factors: contains datablock(s) I. DOI: 10.1107/S1600536812040342/gg2100Isup2.hkl

Supplementary material file. DOI: 10.1107/S1600536812040342/gg2100Isup3.cml

Additional supplementary materials:  crystallographic information; 3D view; checkCIF report

## Figures and Tables

**Table 1:** Hydrogen-bond geometry (Å, °)

*D*—H⋯*A*	*D*—H	H⋯*A*	*D*⋯*A*	*D*—H⋯*A*
O1—H1⋯O2	0.94 (3)	2.52 (2)	3.156 (2)	124.8 (18)
O1—H1⋯N	0.94 (3)	1.79 (3)	2.6352 (19)	147.8 (19)
O2—H2⋯O1^i^	0.88 (3)	1.84 (3)	2.708 (2)	168 (3)

Symmetry code: (i) 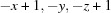.

**Fig. 1.**
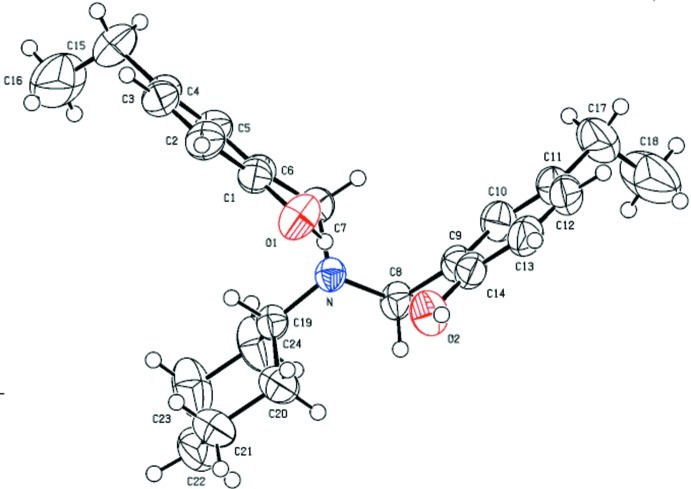
Molecular structure of the title compound (arbitrary spheres for the H atoms).

## supplementary crystallographic information

### Experimental

The preparation of the title compound was reported elsewhere (Laobuthee *et al.*, 2003). Colorless blocks were recrystallized from 2-propanol solution.

### Refinement

All H atoms of the compound were placed in the calculated positions with C—H = 0.96 Å and included in the final cycles of refinement in a rigiding model, *U*_iso_(H) = 1.2 *U*_eq_(H). Except H atom of O atoms were located in different fourier map and restrained to their hosts.

### Crystal data

**Table:**

C_24_H_33_NO_2_	*Z* = 2
*M**_r_* = 367.51	*F*(000) = 400.0
Triclinic, *P*1	*D*_x_ = 1.117 Mg m^−^^3^
Hall symbol: -P 1	Mo *K*α radiation, λ = 0.71073 Å
*a* = 9.4224 (12) Å	Cell parameters from 3314 reflections
*b* = 10.3799 (16) Å	θ = 2.6–28.2°
*c* = 11.8143 (18) Å	µ = 0.07 mm^−^^1^
α = 82.140 (4)°	*T* = 296 K
β = 73.960 (4)°	Block, colourless
γ = 81.676 (4)°	0.90 × 0.44 × 0.24 mm
*V* = 1093.1 (3) Å^3^	

### Data collection

**Table:**

Bruker APEXII CCD diffractometer	3648 reflections with *I* > 2σ(*I*)
Radiation source: fine-focus sealed tube	*R*_int_ = 0.022
Graphite monochromator	θ_max_ = 28.2°, θ_min_ = 1.8°
φ and ω scans	*h* = −12→12
8721 measured reflections	*k* = −13→13
5350 independent reflections	*l* = −15→15

### Refinement

**Table:**

Refinement on *F*^2^	Primary atom site location: structure-invariant direct methods
Least-squares matrix: full	Secondary atom site location: difference Fourier map
*R*[*F*^2^ > 2σ(*F*^2^)] = 0.060	Hydrogen site location: inferred from neighbouring sites
*wR*(*F*^2^) = 0.185	H atoms treated by a mixture of independent and constrained refinement
*S* = 1.12	*w* = 1/[σ^2^(*F*_o_^2^) + (0.085*P*)^2^ + 0.1411*P*] where *P* = (*F*_o_^2^ + 2*F*_c_^2^)/3
5350 reflections	(Δ/σ)_max_ < 0.001
252 parameters	Δρ_max_ = 0.27 e Å^−^^3^
0 restraints	Δρ_min_ = −0.23 e Å^−^^3^

### Special details

**Table:**

Geometry. All e.s.d.'s (except the e.s.d. in the dihedral angle between two l.s. planes) are estimated using the full covariance matrix. The cell e.s.d.'s are taken into account individually in the estimation of e.s.d.'s in distances, angles and torsion angles; correlations between e.s.d.'s in cell parameters are only used when they are defined by crystal symmetry. An approximate (isotropic) treatment of cell e.s.d.'s is used for estimating e.s.d.'s involving l.s. planes.
Refinement. Refinement of *F*^2^ against ALL reflections. The weighted *R*-factor *wR* and goodness of fit *S* are based on *F*^2^, conventional *R*-factors *R* are based on *F*, with *F* set to zero for negative *F*^2^. The threshold expression of *F*^2^ > σ(*F*^2^) is used only for calculating *R*-factors(gt) *etc*. and is not relevant to the choice of reflections for refinement. *R*-factors based on *F*^2^ are statistically about twice as large as those based on *F*, and *R*- factors based on ALL data will be even larger.

### Fractional atomic coordinates and isotropic or equivalent isotropic displacement parameters (Å^2^)

**Table:**

	*x*	*y*	*z*	*U*_iso_*/*U*_eq_	
N	0.15859 (13)	0.23370 (12)	0.54691 (10)	0.0404 (3)	
O1	0.42889 (14)	0.18278 (12)	0.57669 (13)	0.0633 (4)	
O2	0.32304 (15)	−0.01044 (13)	0.44034 (11)	0.0627 (4)	
C6	0.29872 (16)	0.39939 (15)	0.58608 (13)	0.0436 (3)	
C8	0.09657 (17)	0.19375 (17)	0.45693 (14)	0.0487 (4)	
H8A	0.0189	0.2606	0.4417	0.058*	
H8B	0.0520	0.1132	0.4875	0.058*	
C9	0.21273 (17)	0.17318 (17)	0.34271 (14)	0.0479 (4)	
C19	0.06585 (15)	0.21269 (15)	0.67028 (13)	0.0432 (3)	
H19A	0.1157	0.2486	0.7196	0.052*	
C5	0.2872 (2)	0.52251 (17)	0.62205 (16)	0.0560 (4)	
H5A	0.2120	0.5852	0.6064	0.067*	
C1	0.41114 (16)	0.30665 (16)	0.61073 (14)	0.0468 (4)	
C2	0.50701 (18)	0.3377 (2)	0.67020 (17)	0.0594 (5)	
H2A	0.5813	0.2750	0.6872	0.071*	
C14	0.32556 (18)	0.06974 (17)	0.33839 (15)	0.0509 (4)	
C10	0.2111 (2)	0.2556 (2)	0.23952 (16)	0.0619 (5)	
H10A	0.1357	0.3246	0.2419	0.074*	
C13	0.4330 (2)	0.0513 (2)	0.23316 (17)	0.0639 (5)	
H13A	0.5090	−0.0171	0.2303	0.077*	
C4	0.3830 (2)	0.55644 (19)	0.68038 (17)	0.0634 (5)	
C3	0.4923 (2)	0.4615 (2)	0.70427 (17)	0.0634 (5)	
H3A	0.5574	0.4816	0.7442	0.076*	
C12	0.4272 (2)	0.1345 (2)	0.13275 (18)	0.0745 (6)	
H12A	0.4992	0.1200	0.0625	0.089*	
C11	0.3180 (3)	0.2386 (2)	0.13297 (17)	0.0729 (6)	
C20	0.0648 (3)	0.0674 (2)	0.71237 (18)	0.0735 (6)	
H20A	0.1661	0.0253	0.6976	0.088*	
H20B	0.0130	0.0273	0.6683	0.088*	
C24	−0.0908 (2)	0.2822 (3)	0.69530 (19)	0.0811 (7)	
H24A	−0.1468	0.2483	0.6505	0.097*	
H24B	−0.0878	0.3750	0.6708	0.097*	
C21	−0.0116 (3)	0.0471 (2)	0.8443 (2)	0.0883 (7)	
H21A	−0.0157	−0.0457	0.8684	0.106*	
H21B	0.0460	0.0794	0.8887	0.106*	
C15	0.3654 (3)	0.6929 (2)	0.7176 (3)	0.0950 (8)	
H15A	0.4601	0.7105	0.7263	0.114*	
H15B	0.3409	0.7553	0.6550	0.114*	
C23	−0.1674 (2)	0.2610 (3)	0.8282 (2)	0.0953 (9)	
H23A	−0.1171	0.3032	0.8719	0.114*	
H23B	−0.2694	0.3015	0.8428	0.114*	
C17	0.3115 (4)	0.3322 (3)	0.0225 (2)	0.1067 (9)	
H17A	0.2822	0.4205	0.0453	0.128*	
H17B	0.4102	0.3302	−0.0312	0.128*	
C22	−0.1661 (3)	0.1168 (3)	0.8725 (2)	0.0993 (9)	
H22A	−0.2087	0.1074	0.9575	0.119*	
H22B	−0.2272	0.0769	0.8362	0.119*	
C16	0.2541 (4)	0.7143 (3)	0.8259 (3)	0.1463 (15)	
H16A	0.2500	0.8027	0.8432	0.219*	
H16B	0.2785	0.6548	0.8892	0.219*	
H16C	0.1592	0.6998	0.8178	0.219*	
C18	0.2088 (4)	0.3018 (5)	−0.0399 (3)	0.1597 (18)	
H18A	0.2107	0.3637	−0.1083	0.240*	
H18B	0.1100	0.3064	0.0118	0.240*	
H18C	0.2379	0.2151	−0.0641	0.240*	
C7	0.20041 (18)	0.36794 (15)	0.51474 (15)	0.0490 (4)	
H7A	0.1110	0.4295	0.5272	0.059*	
H7B	0.2520	0.3783	0.4313	0.059*	
H1	0.347 (3)	0.176 (2)	0.548 (2)	0.107 (9)*	
H2	0.398 (3)	−0.073 (3)	0.430 (2)	0.095 (8)*	

### Atomic displacement parameters (Å^2^)

**Table:**

	*U*^11^	*U*^22^	*U*^33^	*U*^12^	*U*^13^	*U*^23^
N	0.0376 (6)	0.0464 (7)	0.0397 (6)	−0.0025 (5)	−0.0143 (5)	−0.0062 (5)
O1	0.0465 (6)	0.0623 (8)	0.0889 (10)	0.0105 (5)	−0.0289 (6)	−0.0287 (7)
O2	0.0614 (8)	0.0638 (8)	0.0573 (8)	0.0116 (6)	−0.0139 (6)	−0.0103 (6)
C6	0.0435 (8)	0.0465 (8)	0.0403 (8)	−0.0091 (6)	−0.0092 (6)	−0.0023 (6)
C8	0.0416 (8)	0.0629 (10)	0.0467 (8)	−0.0035 (7)	−0.0193 (6)	−0.0096 (7)
C9	0.0465 (8)	0.0600 (10)	0.0430 (8)	−0.0093 (7)	−0.0169 (7)	−0.0105 (7)
C19	0.0360 (7)	0.0535 (9)	0.0419 (8)	−0.0032 (6)	−0.0117 (6)	−0.0092 (6)
C5	0.0613 (10)	0.0481 (9)	0.0579 (10)	−0.0094 (8)	−0.0136 (8)	−0.0038 (8)
C1	0.0371 (7)	0.0554 (9)	0.0481 (9)	−0.0062 (6)	−0.0086 (6)	−0.0098 (7)
C2	0.0418 (8)	0.0740 (12)	0.0662 (11)	−0.0100 (8)	−0.0165 (8)	−0.0117 (9)
C14	0.0499 (9)	0.0589 (10)	0.0484 (9)	−0.0097 (7)	−0.0137 (7)	−0.0156 (7)
C10	0.0668 (11)	0.0746 (12)	0.0518 (10)	−0.0144 (9)	−0.0263 (9)	−0.0028 (9)
C13	0.0523 (10)	0.0784 (13)	0.0632 (12)	−0.0129 (9)	−0.0055 (8)	−0.0291 (10)
C4	0.0730 (12)	0.0642 (11)	0.0548 (10)	−0.0314 (10)	−0.0042 (9)	−0.0128 (8)
C3	0.0548 (10)	0.0844 (14)	0.0581 (11)	−0.0291 (10)	−0.0136 (8)	−0.0114 (9)
C12	0.0691 (12)	0.1093 (18)	0.0493 (11)	−0.0365 (12)	−0.0006 (9)	−0.0245 (11)
C11	0.0801 (14)	0.1002 (16)	0.0462 (10)	−0.0369 (12)	−0.0178 (9)	−0.0033 (10)
C20	0.0908 (15)	0.0628 (12)	0.0577 (11)	−0.0216 (10)	0.0021 (10)	−0.0050 (9)
C24	0.0465 (10)	0.128 (2)	0.0611 (12)	0.0191 (11)	−0.0117 (9)	−0.0200 (12)
C21	0.1108 (19)	0.0852 (16)	0.0598 (13)	−0.0401 (14)	0.0038 (12)	0.0003 (11)
C15	0.122 (2)	0.0749 (15)	0.0982 (18)	−0.0377 (14)	−0.0226 (16)	−0.0284 (13)
C23	0.0506 (11)	0.163 (3)	0.0645 (13)	0.0154 (14)	−0.0041 (9)	−0.0361 (16)
C17	0.130 (2)	0.145 (3)	0.0532 (13)	−0.0617 (19)	−0.0274 (14)	0.0172 (14)
C22	0.0791 (16)	0.167 (3)	0.0577 (13)	−0.0605 (17)	0.0003 (11)	−0.0198 (15)
C16	0.151 (3)	0.111 (2)	0.162 (3)	−0.030 (2)	0.020 (3)	−0.077 (2)
C18	0.156 (3)	0.253 (5)	0.091 (2)	−0.105 (3)	−0.064 (2)	0.063 (3)
C7	0.0528 (9)	0.0464 (9)	0.0505 (9)	−0.0030 (7)	−0.0218 (7)	−0.0001 (7)

### Geometric parameters (Å, º)

**Table:**

N—C7	1.474 (2)	C12—C11	1.380 (3)
N—C8	1.4756 (19)	C12—H12A	0.9300
N—C19	1.4833 (19)	C11—C17	1.527 (3)
O1—C1	1.373 (2)	C20—C21	1.525 (3)
O1—H1	0.94 (3)	C20—H20A	0.9700
O2—C14	1.364 (2)	C20—H20B	0.9700
O2—H2	0.88 (3)	C24—C23	1.536 (3)
C6—C5	1.381 (2)	C24—H24A	0.9700
C6—C1	1.390 (2)	C24—H24B	0.9700
C6—C7	1.506 (2)	C21—C22	1.498 (4)
C8—C9	1.503 (2)	C21—H21A	0.9700
C8—H8A	0.9700	C21—H21B	0.9700
C8—H8B	0.9700	C15—C16	1.433 (4)
C9—C10	1.393 (2)	C15—H15A	0.9700
C9—C14	1.393 (2)	C15—H15B	0.9700
C19—C24	1.515 (2)	C23—C22	1.516 (4)
C19—C20	1.522 (2)	C23—H23A	0.9700
C19—H19A	0.9800	C23—H23B	0.9700
C5—C4	1.384 (3)	C17—C18	1.458 (4)
C5—H5A	0.9300	C17—H17A	0.9700
C1—C2	1.382 (2)	C17—H17B	0.9700
C2—C3	1.375 (3)	C22—H22A	0.9700
C2—H2A	0.9300	C22—H22B	0.9700
C14—C13	1.386 (2)	C16—H16A	0.9600
C10—C11	1.393 (3)	C16—H16B	0.9600
C10—H10A	0.9300	C16—H16C	0.9600
C13—C12	1.377 (3)	C18—H18A	0.9600
C13—H13A	0.9300	C18—H18B	0.9600
C4—C3	1.379 (3)	C18—H18C	0.9600
C4—C15	1.514 (3)	C7—H7A	0.9700
C3—H3A	0.9300	C7—H7B	0.9700
			
C7—N—C8	111.05 (12)	C21—C20—H20B	109.5
C7—N—C19	112.92 (12)	H20A—C20—H20B	108.1
C8—N—C19	114.48 (11)	C19—C24—C23	109.84 (17)
C1—O1—H1	106.7 (16)	C19—C24—H24A	109.7
C14—O2—H2	111.5 (16)	C23—C24—H24A	109.7
C5—C6—C1	117.94 (15)	C19—C24—H24B	109.7
C5—C6—C7	121.62 (15)	C23—C24—H24B	109.7
C1—C6—C7	120.30 (14)	H24A—C24—H24B	108.2
N—C8—C9	112.25 (12)	C22—C21—C20	111.5 (2)
N—C8—H8A	109.2	C22—C21—H21A	109.3
C9—C8—H8A	109.2	C20—C21—H21A	109.3
N—C8—H8B	109.2	C22—C21—H21B	109.3
C9—C8—H8B	109.2	C20—C21—H21B	109.3
H8A—C8—H8B	107.9	H21A—C21—H21B	108.0
C10—C9—C14	118.44 (16)	C16—C15—C4	114.7 (2)
C10—C9—C8	121.57 (16)	C16—C15—H15A	108.6
C14—C9—C8	119.99 (15)	C4—C15—H15A	108.6
N—C19—C24	116.08 (14)	C16—C15—H15B	108.6
N—C19—C20	111.12 (13)	C4—C15—H15B	108.6
C24—C19—C20	110.84 (17)	H15A—C15—H15B	107.6
N—C19—H19A	106.0	C22—C23—C24	111.7 (2)
C24—C19—H19A	106.0	C22—C23—H23A	109.3
C20—C19—H19A	106.0	C24—C23—H23A	109.3
C6—C5—C4	122.68 (17)	C22—C23—H23B	109.3
C6—C5—H5A	118.7	C24—C23—H23B	109.3
C4—C5—H5A	118.7	H23A—C23—H23B	107.9
O1—C1—C2	118.84 (15)	C18—C17—C11	113.9 (2)
O1—C1—C6	120.72 (14)	C18—C17—H17A	108.8
C2—C1—C6	120.44 (16)	C11—C17—H17A	108.8
C3—C2—C1	119.93 (18)	C18—C17—H17B	108.8
C3—C2—H2A	120.0	C11—C17—H17B	108.8
C1—C2—H2A	120.0	H17A—C17—H17B	107.7
O2—C14—C13	122.99 (17)	C21—C22—C23	111.59 (18)
O2—C14—C9	117.11 (15)	C21—C22—H22A	109.3
C13—C14—C9	119.90 (17)	C23—C22—H22A	109.3
C11—C10—C9	122.5 (2)	C21—C22—H22B	109.3
C11—C10—H10A	118.8	C23—C22—H22B	109.3
C9—C10—H10A	118.8	H22A—C22—H22B	108.0
C12—C13—C14	119.99 (19)	C15—C16—H16A	109.5
C12—C13—H13A	120.0	C15—C16—H16B	109.5
C14—C13—H13A	120.0	H16A—C16—H16B	109.5
C3—C4—C5	117.73 (17)	C15—C16—H16C	109.5
C3—C4—C15	121.8 (2)	H16A—C16—H16C	109.5
C5—C4—C15	120.5 (2)	H16B—C16—H16C	109.5
C4—C3—C2	121.27 (17)	C17—C18—H18A	109.5
C4—C3—H3A	119.4	C17—C18—H18B	109.5
C2—C3—H3A	119.4	H18A—C18—H18B	109.5
C11—C12—C13	122.14 (18)	C17—C18—H18C	109.5
C11—C12—H12A	118.9	H18A—C18—H18C	109.5
C13—C12—H12A	118.9	H18B—C18—H18C	109.5
C12—C11—C10	117.07 (19)	N—C7—C6	112.32 (12)
C12—C11—C17	122.6 (2)	N—C7—H7A	109.1
C10—C11—C17	120.4 (2)	C6—C7—H7A	109.1
C19—C20—C21	110.63 (17)	N—C7—H7B	109.1
C19—C20—H20A	109.5	C6—C7—H7B	109.1
C21—C20—H20A	109.5	H7A—C7—H7B	107.9
C19—C20—H20B	109.5		
			
C7—N—C8—C9	70.20 (17)	C6—C5—C4—C15	179.91 (18)
C19—N—C8—C9	−160.49 (13)	C5—C4—C3—C2	0.7 (3)
N—C8—C9—C10	−112.40 (16)	C15—C4—C3—C2	179.84 (19)
N—C8—C9—C14	67.57 (19)	C1—C2—C3—C4	0.1 (3)
C7—N—C19—C24	69.65 (18)	C14—C13—C12—C11	1.0 (3)
C8—N—C19—C24	−58.74 (19)	C13—C12—C11—C10	−1.0 (3)
C7—N—C19—C20	−162.49 (14)	C13—C12—C11—C17	179.35 (19)
C8—N—C19—C20	69.13 (18)	C9—C10—C11—C12	0.6 (3)
C1—C6—C5—C4	0.4 (2)	C9—C10—C11—C17	−179.71 (18)
C7—C6—C5—C4	−175.33 (16)	N—C19—C20—C21	171.75 (17)
C5—C6—C1—O1	−179.63 (15)	C24—C19—C20—C21	−57.6 (2)
C7—C6—C1—O1	−3.9 (2)	N—C19—C24—C23	−174.90 (18)
C5—C6—C1—C2	0.5 (2)	C20—C19—C24—C23	57.1 (2)
C7—C6—C1—C2	176.22 (15)	C19—C20—C21—C22	56.1 (3)
O1—C1—C2—C3	179.41 (16)	C3—C4—C15—C16	−97.6 (3)
C6—C1—C2—C3	−0.7 (3)	C5—C4—C15—C16	81.5 (3)
C10—C9—C14—O2	−179.05 (14)	C19—C24—C23—C22	−55.7 (3)
C8—C9—C14—O2	1.0 (2)	C12—C11—C17—C18	97.7 (3)
C10—C9—C14—C13	0.3 (2)	C10—C11—C17—C18	−81.9 (4)
C8—C9—C14—C13	−179.66 (14)	C20—C21—C22—C23	−54.7 (3)
C14—C9—C10—C11	−0.3 (3)	C24—C23—C22—C21	54.8 (3)
C8—C9—C10—C11	179.68 (16)	C8—N—C7—C6	−165.28 (12)
O2—C14—C13—C12	178.65 (16)	C19—N—C7—C6	64.58 (16)
C9—C14—C13—C12	−0.7 (3)	C5—C6—C7—N	−143.79 (15)
C6—C5—C4—C3	−1.0 (3)	C1—C6—C7—N	40.6 (2)

### Hydrogen-bond geometry (Å, º)

**Table:**

*D*—H···*A*	*D*—H	H···*A*	*D*···*A*	*D*—H···*A*
O1—H1···O2	0.94 (3)	2.52 (2)	3.156 (2)	124.8 (18)
O1—H1···N	0.94 (3)	1.79 (3)	2.6352 (19)	147.8 (19)
O2—H2···O1^i^	0.88 (3)	1.84 (3)	2.708 (2)	168 (3)

Symmetry code: (i) −*x*+1, −*y*, −*z*+1.
